# Antifungal Activity and Alleviation of Salt Stress by Volatile Organic Compounds of Native *Pseudomonas* Obtained from *Mentha piperita*

**DOI:** 10.3390/plants12071488

**Published:** 2023-03-29

**Authors:** Samanta Soledad Gil, Lorena del Rosario Cappellari, Walter Giordano, Erika Banchio

**Affiliations:** INBIAS Instituto de Biotecnología Ambiental y Salud (CONICET—Universidad Nacional de Río Cuarto), Campus Universitario, Río Cuarto 5800, Argentina

**Keywords:** microbial volatile organic compound, mVOCs, plant-growth-promoting rhizobacteria (PGPR), *Mentha piperita*, salt stress, *Pseudomona putida*, biocontrol, phytopathogenic fungi

## Abstract

As salt stress has a negative impact on plant growth and crop yield, it is very important to identify and develop any available biotechnology which can improve the salt tolerance of plants. Inoculation with plant-growth-promoting rhizobacteria (PGPR) is a proven environmentally friendly biotechnological resource for increasing the salt stress tolerance of plants and has a potential in-field application. In addition, bacterial volatile organic compounds (mVOCs) are signal molecules that may have beneficial roles in the soil–plant–microbiome ecosystem. We investigated the effects of mVOCs emitted by *Pseudomona putida* SJ46 and SJ04 on *Mentha piperita* grown under different levels of NaCl stress by evaluating their growth-promoting potential and capacity to increase salt tolerance effects. Furthermore, we evaluated under control and salt stress conditions the biocontrol ability of VOCs emitted by both these strains to inhibit the growth of *Alternaria alternata* and *Sclerotium rolfsii*. The VOCs emitted by both strains under control conditions did not lead to an significant improvement in peppermint growth. However, under salt stress conditions (75 or 100 mM NaCl), an amelioration of its physiological status was observed, with this effect being greater at 100 mM NaCl. This led to an enhancement of the number of leaves and nodes and, increased the shoot fresh and root dry weight by approximately twice in relation to control stressed plants. Moreover, the VOCs released by the two bacteria grown in control or saline media showed a significant reduction in the mycelial growth of *A. alternata*. In contrast, *S. rolfsii* growth was reduced 40% by the mVOCs released only under control conditions, with no effects being observed under salt stress. We also explored the composition of the bacterial volatile profiles by means of a solid-phase microextraction/gas chromatography–mass spectrometry (SPME/GC–MS) analysis. From the headspace of SJ46, three VOCs were identified: n-octanol, decane and tetradecane. The emission of SJ04 had the same chromatographic profile, with the addition of two more compounds: 1-(N-phenyl carbamyl)-2-morpholino cyclohexene and tridecane. Only compounds that were not present in the headspace of the control groups were recorded. The salt stress conditions where the bacteria were grown did not qualitatively modify the mVOC emissions. Taken together, our results suggest that plant-associated rhizobacterial VOCs play a potentially important role in modulating plant salt tolerance and reducing fungal growth. Thus, biological resources represent novel tools for counteracting the deleterious effects of salt stress and have the potential to be exploited in sustainable agriculture. Nevertheless, future studies are necessary to investigate technological improvements for bacterial VOC application under greenhouse and open field conditions.

## 1. Introduction

According to the Food and Agriculture Organization of the United Nations (FAO), incorrect land management and various detrimental environmental factors reduce agricultural productive areas by 1–2% each year, a decrease that is expected to accelerate in the coming decades [1]. Salinity is a soil condition characterized by high concentrations of soluble salts, which is a very important stress factor for plants [2,3]. In general, salinity impacts negatively on the morphological and biochemical functions of plants, inhibiting seed germination, growth, development, and plant yield [4,5]. The deleterious effects of sodium (Na^+^) on plants are associated with the excessive accumulation of ions in the root zone altering the soil texture, reducing osmotic potential, porosity, water conductance and aeration, and increasing the water retention capacity and osmotic pressure. These effects restrict the absorption of water and important ions, and furthermore, the ionic imbalances induced affect the absorption of nutrients by plants [6,7,8]. Under conditions of excess Na^+^, the levels of growth regulators are altered, decreasing the protein synthesis and impairing the photosynthetic processes [9,10]. Another effect of salt stress is oxidative damage, which is caused by the accumulation of reactive oxygen species (ROS); these are known to be detrimental to cells at high concentrations [11,12,13], and if the intracellular concentration of ROS is not controlled, the direct consequences are damage to the cell structure by lipid peroxidation, protein oxidation, nucleic acid damage and, enzymatic inhibition, ultimately leading to cell death [14,15,16].

Microorganisms play a vital role in the agricultural field as they are important for reducing the harmful effects of abiotic stress (drought, high and low temperatures, salinity, metal toxicity, etc.) in crop production [17]. The promotion of growth in plants produced by PGPR can be multifactorial, such as resulting from the solubilization of phosphates, production of siderophores, biological fixation of nitrogen, production of the enzyme 1-Aminocyclopropane 1-carboxylic acid (ACC) deaminase, production and regulation of phytohormones, biocontrol activity, production of volatile organic compounds (VOCs), and the activation of induced systemic resistance (ISR) [18].

In recent years, there has been increased interest in studying VOCs released by PGPR [19,20,21,22,23,24]. Many types of soil bacteria can emit mVOCs, although the type and quantity of the compounds released may differ between species and culture conditions [25,26]. mVOCs are secondary metabolites, mainly derived from fermentation reactions, such as aldehydes, ketones, alcohols, and hydrocarbons [27,28,29,30,31]. They are characterized by being of low molecular weight and having a high vapor pressure and a low boiling point [32,33], with these characteristics being favorable for their evaporation and diffusion through air, water, soil and rhizospheric environments [34,35,36].

Numerous investigations have demonstrated the involvement of VOCs produced by rhizobacteria in the promotion of plant growth [37,38] or in the activation of ISR in plants through the recognition of molecular patterns and the triggering of its defense machinery [38,39,40]. These compounds also participate in the direct inhibition of pathogens through their negative effect on the growth of the mycelium or spores, or by reducing the motility and biofilm formation [41,42].

*Mentha piperita* L. (peppermint) is a hybrid (*M. aquatic* × *M. spicata*) that is an important medicinal and aromatic herb worldwide. It is a rich source of secondary metabolites such as terpenoids and polyphenols, which have strong biological effects [43]. In recent years, the demand for *Mentha piperita* L. has increased, mainly due to changes in consumption habits seeing an increase in pre-prepared foods and with greater demand for condiments with antioxidant properties. This trend towards a healthier life has simulated a desire for flavorings, supplements and preservatives of natural origin [44], and for the development of bioactive compounds, which are used as a tool to complement and/or replace chemically synthesized compounds in current production models [45].

Peppermint plants are negatively affected by salinity-affected soil, which severely decreases the biomass and essential oil yield [46]. Therefore, different techniques have been proposed to mitigate the negative effects of salt stress in peppermint crops. These approaches include the use of salt-tolerant varieties, stress signaling molecules, osmoprotectants, green algae and plant extracts, with plant and green algae extract having demonstrated a great deal of promise for crop enhancement in moderate stress conditions in recent years [47]. It is still necessary, however, to look for new alternatives to minimize this problem since the amount of soils affected by salinity is increasing.

In previous studies, we have shown that direct inoculation of native *P. putida* SJ04 produces beneficial effects on *M. piperita*, including growth promotion and increased essential oil yield, plant VOC emission and total phenolic compounds [48,49,50]. Furthermore, SJ04 mVOCs induce changes in the essential oil composition [51]. Taking into account these properties of direct inoculation, as well as VOC emission from *P. putida* native to the *M. piperita* rhizosphere, we proposed to determine their capability for helping plants to cope with adverse salt stress conditions. The study of volatile organic compounds of microbial origin is still a little explored field, so the specific effects produced by the volatile compounds in the plant need also to be evaluated under abiotic conditions. The *Arabidopsis thaliana* plant has been widely used as a study model in plant–microorganism interactions. However, it is necessary to analyze other models in order to determine whether these discoveries can be applied more generally in the plant kingdom, since there is a possibility that the mechanisms may be different [52]. Therefore, the present study was carried out using the aromatic and medicinal plant species *M. piperita*. Thus, the present survey was designed to analyze the bioactivity of mVOCs emitted by *P. putida* grown under control and salt stress conditions as a biocontrol of phytopathogenic fungi and as a plant growth promoter: (i) exploring whether VOCs produced by native *P. putida* under salt stress conditions affect the tolerance of peppermint plants; (ii) determining if mVOCs produced by *P. putida* under salt stress conditions affect the growth of major fungal phytopathogens; and (iii) verifying whether the salt condition of growth media affects the VOC emissions of *P. putida.*

## 2. Results

### 2.1. Plant-Growth-Promoting Effects of mVOCs under Salinity Conditions

When plants were exposed to mVOCs under control conditions, only the shoot length was increased (*p* < 0.05) by both strains (Table 1). In contrast, when plants were grown under 75 and 100 mM salt stress conditions, the SJ46 mVOCs increased the number of leaves significantly, by approximately 60–70% (*p* < 0.05), while SJ04 only showed an increase at 100 mM. The number of ramifications revealed the same tendency for both stress conditions for SJ46 mVOCs, but with SJ04 only showing an increase at 100 mM (*p* < 0.05). The number of nodes was increased by mVOCs of both strains under all conditions evaluated (*p* < 0.05). However, only SJ46 mVOCs increased the shoot length significantly under 100 mM conditions (*p* < 0.05).

**Figure 1:**
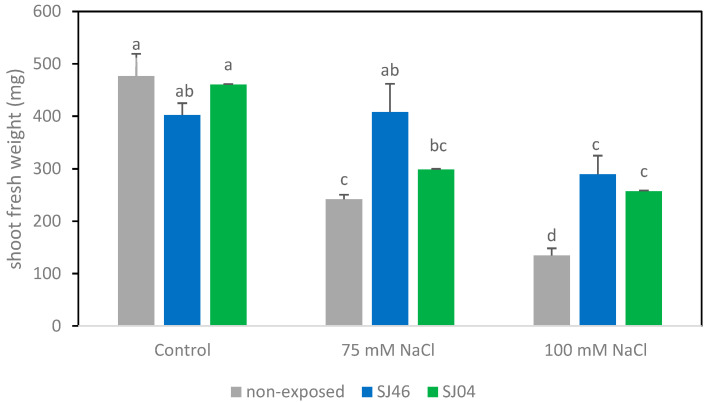
Effects of exposure to SJ46 and SJ04 mVOCs of *P. putida* on shoot fresh weight of *M. piperita* plants grown in Murashige–Skoog medium with 0, 75 and 100 mM NaCl. Values are mean ± standard error (SE). Means followed by the same letter in a given column are not significantly different according to Fisher’s LSD test (*p* < 0.05).

Regarding the fresh weight of the shoot, a negative effect of salt (*p* < 0.05) was clearly observed, with plants grown under 75 and 100 mM registering just a half and a third, respectively, of the shoot fresh weight of control plants (Figure 1). When plants were exposed to mVOCs in the absence of salt stress (0 mM), none of the strains evaluated (SJ04 and SJ46) revealed growth-promoting effects (*p* > 0.05). The opposite was observed at 75 mM NaCl, as compared to control plants (not exposed to mVOCs); the shoot fresh weight increased by approximately 70% when exposed to SJ46 VOCs. The same tendency was observed for severe stress (100 mM), with an increase of approximately two times being recorded in plants in contact with SJ04 or SJ46 volatiles, compared to the control without VOC exposure (*p* < 0.05). The weight values of plants grown under 100 mM and treated with mVOC emissions were similar to untreated plants under 75 mM NaCl.

**Figure 2:**
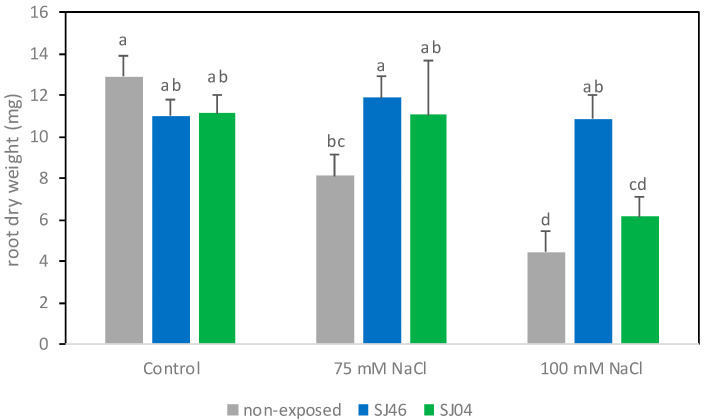
Effects of exposure to SJ46 and SJ04 mVOCs of *P. putida* on root dry weight of *M. piperita* plants grown in Murashige–Skoog medium with 0, 75 and 100 mM NaCl. Values are mean ± standard error (SE). Means followed by the same letter in a given column are not significantly different according to Fisher’s LSD test (*p* < 0.05).

Under non-salt stress conditions, the mVOCs did not significantly affect the root dry weight (Figure 2) or the length of the main root (Table 1) (*p* > 0.05). Under 75 mM, the root dry weight from plants treated with SJ04 or SJ46 mVOCs showed a 35–45% increase, respectively, in comparison with plants stressed but not exposed to mVOCs (*p* < 0.05). Under 100 mM conditions, only plants exposed to SJ46 showed an increase, of approximately 2.5 times, in comparison to the control (not exposed to mVOCs under exposure 100 mM). For both stress conditions, the root dry weight of plants treated with SJ46 was similar to plants grown under non-salt stress conditions. The root length was negatively affected by salt stress (*p* < 0.05), but the mVOCs did not modify the root length in either control conditions or under salt stress (*p* < 0.05) (Table 1).

### 2.2. Effect of Bacterial VOCs on the Growth of Phytopathogen Fungus

**Figure 3:**
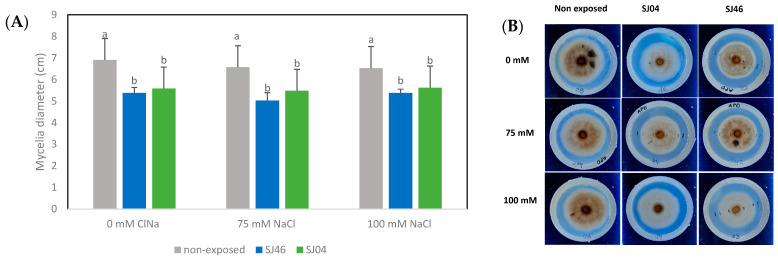
(**A**) Effect of the mVOCs of two PGPR, *P. putida* SJ04 and SJ46, grown under salt stress conditions (0 mM, 75 mM and 100 mM) on the growth of *A. alternata* on the sixth day of the challenge. (**B**) Photos of *A. alternata* cultures exposed to different treatments. Different letters indicate statistically significant differences. Fisher’s LSD test (*p* < 0.05).

The mVOCs produced by SJ46 and SJ04 of *P. putida* reduced the mycelial growth of *A. alternata* by approximately 20%, after 6 days of exposure, for the different conditions assessed (0, 75 and 100 mM NaCl) (*p* < 0.05) (Figure 3).

**Figure 4:**
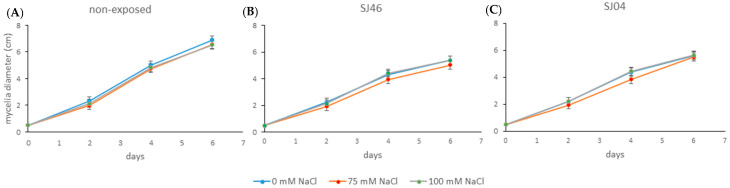
Effect of mVOCs of (**A**) control (non-exposed), (**B**) *P. putida* SJ46 and (**C**) SJ04 on *A. alternata* growing under different salt levels.

It was observed that the mycelial growth of *A. alternata* when grown under non-exposed conditions to the mVOCs at different salt concentrations (0, 75 and 100 mM) at 2, 4 and 6 days did not cause any significant differences (*p* > 0.05) (Figure 4A), with the same tendency being observed when exposed to SJ46 or SJ04 mVOC emissions grown under different salt levels (Figure 4B,C).

**Figure 5:**
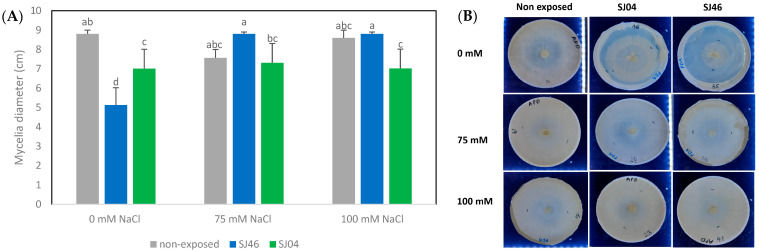
(**A**) Effect of the mVOCs of two PGPR, *P. putida* SJ46 and SJ04, grown under salt stress conditions (0 mM, 75 mM and 100 mM) on the growth of *Sclerotium rolfsii* on the fourth day of the challenge. (**B**) Photos of *S. rolfsii* cultures exposed to different treatments. Different letters indicate statistically significant differences. Fisher’s LSD test (*p* < 0.05).

The radial growth of the *S. rolfsii* colony after 4 days of exposure to SJ46 and SJ04 mVOCs under control conditions (0 mM) reached an inhibition rate of approximately 40% and 20%, respectively (*p* < 0.05) (Figure 5). Exposure to mVOCs emitted by SJ46 and SJ04 grown under 75 and 100 mM NaCl did not modify the mycelia diameter of *S. rolfsii* (*p* > 0.05).

**Figure 6:**
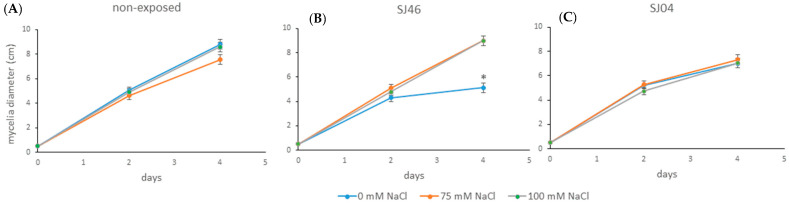
Effect of mVOCs of (**A**) control (non-exposed), (**B**) *P. putida* SJ46 and (**C**) SJ04 on *S. rolfsii* growing under different salt levels. “*” above the columns indicates a significant difference at *p* < 0.05.

The VOCs emitted by SJ46 and SJ04 under salt concentrations had different effects on the growth of *S. rolfsii* during the 4 days of the assay (Figure 6). Under 0, 75 and 100 mM, the VOC emissions of non-bacterial and SJ04 did not modify the growth of *S. rolfsii* on days 2 or 4 (*p* > 0.05) (Figure 6A,C). However, the VOCs emitted by SJ46 under salt stress (75 or 100 mM) increased the mycelium growth of *S. rolfsii*, but this was only observed on the fourth day (*p* < 0.05) (Figure 6B).

### 2.3. Chemical Analysis of mVOCs

Due to the observed positive effect of VOCs emitted by SJ46 and SJ04 on *M. piperita* grown under salt stress conditions and also the inhibitory effect on phytopathogenic fungi, a headspace analysis was carried out to identify the mVOCs produced by non-inoculated vials, DH5 α, *P. putida* SJ04, and SJ46. Based on the chromatographic profiles obtained for the strains under different salt stress conditions (0, 75 and 100 mM) (Supplementary Figures S1–S4), the compounds shown in Table 2 were identified. It was observed that salt conditions did not modify the compounds found in the emission blend of the different strains evaluated. It was possible to recognize the volatile compounds emitted by the culture media (Hoagland’s medium) and by DH5α (which were present in both the control and the treatments) and also the compounds present only in the headspace of both strains SJ04 and SJ46 (Table 2). In the headspace of SJ04, a total of five components were identified, belonging to three classes: hydrocarbons (decane, tridecane, tetradecane), alcohol (n-octanol), and aromatic compounds (1-(N-phenyl carbamyl)-2-morpholinocyclohexene). The headspace of SJ46 had the same chromatographic profile, with the exception that two compounds, (1-(N-phenyl carbamyl)-2-morpholino cyclohexene and tridecane, were not detected (Table 2 and Supplementary Figure S4).

## 3. Discussion

Plant growth parameters are frequently reduced in response to salt stress conditions [53]. However, some studies have reported that mVOCs can induce tolerance against abiotic stress [25,54,55,56]. In the present study, we observed that the growth parameters were reduced in peppermint plants subjected to saline stress, but this negative effect was reversed for plants grown under 75 mM NaCl when exposed to SJ46 VOCs, and also for those grown under 100 mM NaCl when exposed to either SJ04 or SJ46 mVOCs. In these cases, the number of leaves and the shoot weight and length were increased, as well as the root development, with a corresponding gain in root dry weight.

An enhanced root system increases the ability of plants to uptake water from the surroundings [57]. Moreover, increasing root development may have the ecological consequence of increasing colonization of roots by beneficial rhizobacteria [58]. Some related effects were previously observed in *M. piperita* exposed to *Bacillus amyloliquefaciens* GB03 VOCs and grown under 75 and 100 mM, with six compounds being identified in the GB03 mVOCs, of which acetoin (3-hydroxybutanone) was the main compound present in the GB03 blend [54]. In agreement, Ledger et al. [59] showed that VOCs emitted by *Paraburkholderia phytofirmans* mitigated salt stress effects in *Arabidopsis thaliana*, increasing the primary root length, rosette area and total fresh weight under 150 mM NaCl salt stress. Related to this, it has been shown by transcriptional analysis that gene expression is modified in the presence of salinity in *A. thaliana* in the case of the transporter of K^+^ (HKT1), which is responsible for the adjustment of the Na^+^ and K^+^ levels. Moreover, the expression of the HKT1 gene is decreased, thereby reducing the Na^+^ influx through the root. Transcriptional analysis has also shown that VOCs produced by GB03 [60,61], such as 2,3-butanediol [25], decreased the expression of HKT1 in the root, but increased it in the aerial part, thus maintaining the balance in Na^+^ levels throughout the plant [62]. In addition, GB03 VOCs positively affected *A. thaliana* under salt stress by promoting the accumulation of the osmoprotectants of choline and glycine betaine [63]. A similar observation was made in soybean plants grown under salt stress, where VOCs emitted by *Pseudomonas simiae* decreased Na^+^ concentrations and increased phosphorus and potassium [64]. This study showed that proline and other vegetative storage proteins that confer salt tolerance increased in the roots of plants exposed to VOCs. Additionally, *P. simiae* VOCs in combination with sodium nitroprusside enhanced salt tolerance [65], revealing an up-regulation of the gene expression of peroxidase, catalase, nitrite reductase, and vegetative storage protein, whereas the HKT1 transporter pyrroline-5-carboxylate synthase and polyphenol oxidase were down-regulated [65]. In another study, the VOCs emitted by *Alcaligenes faecalis* promoted *A. thaliana* grown under salt stress conditions by the regulation of the auxin and gibberellin pathways involved in the modulation of root and shoot growth and development [66], along with the regulation of ion transporters [67]. Moreover, the PGPR *Paraburkholderia phytofirmans* VOCs reduced the accumulation of sodium within leaf tissues. Similarly, 2R-3R-butanediol, produced the closure of the stomata, generating a decrease in water evaporation [68]. mVOCs from *Rahnella aquatilis* JZ-GX1 ameliorated the salt stress effects on acacia seedlings by a decrease in malondialdehyde, superoxide anion and hydrogen peroxide content, and an increase in the proline level, superoxide dismutase, peroxidase and glutathione reductase activities [69].

mVOCs may also play an important role in bacteria through phytopathogenic fungi interaction. There are many reports where PGPR VOC blends have shown antifungal properties against a wide range of phytopathogenic fungi [70], although the mechanisms involved in such processes remain poorly understood. Recent studies have suggested that VOCs affect phytopathogens by modulating the activity of specific enzymes and altering motility and protein production, which subsequently influence growth, cell morphology and virulence factors [53]. VOCs from *Pseudomonas* sp. have been shown to cause DNA damage to the sugarcane pathogen *Thielaviopsis ethacetica* [71]. Structural damage on hyphae and down-regulation in the expression of virulence factors in *Ralstonia solanacearum* are other mechanisms by which VOCs act [72]. In the present study, inhibition of *A. alternata* and *S. rolfsii* growth by mVOCs emitted by both bacterial strains was observed under control conditions (0 mM NaCl). In agreement with our results, it has been shown that some mVOCs produce the inhibition of the vital activity of fungi in the soil, thereby negatively affecting the germination and growth of fungi [73,74]. Ossowicki et al. [75] also showed this effect, when two fungal strains *Rhizoctonia solani* AG2.2IIIB and *Fusarium culmorum* PV were used against the *Pseudomonas donghuensis* strain P482, with the same being reported for the *P. fluorescens* ZX strain against *Penicillium italicum* [76]. However, in the present study, it was observed that the presence of stress did not limit the production of VOCs, but modified the biocontrol capacity. When PGPR SJ46 and SJ04 were grown under salt stress conditions, mVOCs only inhibited the mycelial growth of *A. alternata*, with mVOCs emitted under salt stress not affecting *S. rolfsii* growth, suggesting that the fungi response to mVOCs is species-specific and depends on the concentrations of certain compounds rather than their composition, since we did not find any new compound in the headspace obtained from salt treatments. Moreover, we showed that SJ46 VOC emissions under salt stress conditions (75 or 100 mM NaCl) promoted greater *S. rolfsii* mycelium growth in comparison with SJ46 VOC emissions at 0 mM NaCl (Figure 5). The above results suggest that the same mVOC blends may have different effects on the receptor organism, as in the case of the different fungi used in this study (*A. alternata* and *S. rolfsii*). Similar results were observed by Guevara Avedaño et al. [77], where the mVOCs produced by different avocado rhizobacteria belonging to the genus *Bacillus* were able to suppress the growth of *Fusarium* sp., but not all inhibited *Colletotrichum gloeosporioides*, and none reduced the diameter of the mycelial growth of *Phytophthora cinnamomi*.

The mVOC profiles of the two *P. putida*, SJ04 and SJ46, grown under different salt stress conditions showed that they emit similar compounds. In the headspace of SJ46, considering only the compounds not present in the control profiles, there were three other compounds identified, belonging to hydrocarbon and alcohol classes, with similar VOCs being found in the SJ04 emission with the addition of 1-(N-phenylcarbamyl)-2-morpholinocyclohexene and tridecane. In particular, the hydrocarbon decane identified in the SJ46 and SJ04 mVOCs was previously detected in *Pseudomonas* strain blends but did not display specific biocontrol activity against the plant pathogenic fungus *Sclerotinia sclerotiorum* [78]. In addition, in another study, it was found in the headspace of *B. subtilis* GB03, *B. subtilis* 168, and *B. amyloliquefaciens* IN937a [79], with effects of systemic resistance induction (ISR) having been described in *Arabidopsis*. Tridecane was also identified in *B. pumilus* ES4 emissions, and *Azospirillum brasilense* Cd showed a growth-promoting effect on *Chlorella sorokiniana* [80]. This was also identified in volatiles of *Serratia* sp. [81] and *Paenibacillus* sp. P4 [82]. Both tridecane and tetradecane were detected in *P. simiae* and were found to increase seed germination and higher fresh weight in soybean plants [65]. Octanol was identified in the *B. subtilis* CF-3 VOCs emitted in the blend with 2,4-di-tert-butylphenol and benzothiazole, with these compounds showing significant positive correlations with the rates of *Monilinia fructicola* and *Colletotrichum gloeosporioides* inhibition [83]. Finally, octanol has been demonstrated to completely inhibit spore germination of *Penicillium camemberti* at low concentrations [84].

The VOC profiles of SJ04 and SJ46 were found to be similar, possibly because both rhizobacteria are closely related to each other phenotypically and genotypically, so they were presumably subjected to similar selective pressures since they were obtained from the same place of origin [51,85,86]. In the present study, we only used Hoagland, which is a minimum culture media. In contrast, Heenan-Daly et al. [87] examined the mVOC emissions of six bacteria from the genera *Bacillus*, *Serratia* and *Pseudomonas* in three different media types. These authors found that Murashige and Skoog (M + S) was responsible for a smaller number of mVOCs detected in comparison with methyl red-Voges Proskeur (MR-VP) and tryptic soy broth (TSB), suggesting that these results may have been attributed to the carbon-rich composition of the media. It is also important to mention that the VOC profile emissions detected are conditioned by the different techniques used, with the identification of VOCs being highly dependent on the SPME fiber or the organic solvent used to collect compounds by dynamic headspace. In our study, we analyzed the headspace only by the DVB/CAR/PDMS SPME fiber. However, some studies have revealed some differences in mVOCs when analyzed by three different kinds of extraction fibers, such as 85 μm polyacrylate (PA), 100 μm PDMS, and 7 μm PDMS [83]. In fact, the fiber coating plays a crucial role in adsorbing VOCs of a specific chemical nature, based on the polarity and size [28]. It has been shown that some substances are high in content but their bioactive effect is not obvious, while other substances are low in content, but their effect can be very significant [88]. Even when investigators have evaluated the promoting activity using standards, they have often not observed the same level of response in comparison with the whole VOC blend [53]. In addition, the presence of the main active compounds with bioactive effects on the VOC profile does not always ensure the same response or effect on the receiver organism. Guevara-Avendano et al. [77] reported that the VOC emissions of *Pseudomonas* isolated from avocado rhizobacteria did not reduce the mycelium growth of *Phytophthora cinnamomi*, although dimethyl disulfide (DMDS) and dimethyl trisulfide (DMTS) were identified in the volatile profile. However, it was reported that DMDS and DMTS completely inhibited the mycelial growth of diverse phytopathogen fungi when tested as commercial standards.

Summing up, it is possible to find the same PGPR strain reported in different mVOC profiles and different bioactivities, so it is necessary to consider the different methods used to determine the mVOC profiles and to perform a functional validation of each compound. Furthermore, microorganisms exhibit substantial genetic and metabolic plasticity, which can lead to different responses to those observed in previous tests or tests performed by different research groups [53].

## 4. Materials and Methods

### 4.1. Bacterial Cultures

The two strains SJ04 (GenBank KF312464.1) and SJ46 (GenBank KF312478.1), previously reported as being PGPR and belonging to the species *Pseudomonas putida*, and which had been isolated from the rhizosphere of *Mentha piperita* from a commercial plantation in the Villa Dolores Region, Córdoba, were used for the bacterial cultures [51,89]. These strains were selected based on the results obtained in the determination of the PGPR activities mediated by VOCs in previous studies [51]. *Escherichia coli* DH5α was selected as a negative control since it is a reference strain without PGPR activity and is not present in the soil environment. Stocks were prepared in sterility by adding 0.2 mL of sterile glycerol to 0.8 mL of a bacterial culture grown to the late exponential phase. The bacterial strains were preserved in glycerol stocks at −80 °C.

Strains were grown on LB medium (10 g/L tryptone, 5 g/L yeast extract, 5 g/L NaCl) for routine use. The bacterial culture was grown overnight at 28 °C and rotated at 150 rpm until reaching the exponential phase, after which, it was washed twice in 0.9% NaCl by Eppendorf centrifugation (10,000 rpm, 10 min, 25 °C), re-suspended in sterile water and adjusted to a final concentration of ~10^9^ CFU/mL for use as inoculum.

### 4.2. In Vitro Plant Exposure to mVOCs

A methodology carried out by Cappellari and Banchio [54] was used with some modifications. Individual nodes of grown seedlings were planted in sterilized glass jars (250 mL) containing 40 mL of solid MS medium with 0.7% (*w*/*v*) agar [90] and 3% (*w*/*v*) sucrose. A small glass vial (10 mL) containing 5 mL of Hoagland medium [91] with 0.7% (*w*/*v*) agar and 3% (*w*/*v*) sucrose was placed inside the bottle. Hoagland with added sugar was used for the bacteria since, as it is a minimal medium based on salts, we consider it to closely resemble soil. Other possible similar culture media have complete carbon sources, such as meat extract or yeast extract, but these are not similar to the carbon sources of the soil. The bacterial strains used were previously cultured in LB broth, centrifuged, washed and resuspended in a sterile physiological solution until reaching, at 660 nm, an OD of 1. From this solution, 100 μL aliquots were extracted to inoculate the vials, which also served as a source of bacterial volatiles. As a control, instead of bacterial inoculation, a physiological solution was used. In this way, the plants were exposed to the bacterial VOCs without having any physical contact with the rhizobacteria. The 250 mL glass vials containing plants and bacteria were covered with aluminum foil and sealed with film. Thus, the peppermint explants were exposed to VOCs produced by microorganisms and contamination was avoided. Then, the vials were placed in a growth chamber under controlled conditions of light (16 h/8 h light/dark cycle), temperature (26 ± 2 °C) and relative humidity (~70%). After 45 days, the plants were harvested and the biomass data were recorded. The experiments were then repeated in triplicate (10 jars per treatment; 1 plant per jar).

### 4.3. Salt Treatments

MS media (plant growth media) and Hoagland media (bacterial growth media) were supplemented with salt concentrations of (a) 0 mM NaCl, (b) 75 mM NaCl, or (c) 100 mM NaCl. In each experimental set, both the plant and bacteria were always grown under the same concentration of NaCl.

### 4.4. Plant Growth Measurement

When each plant was removed from its glass jar, its roots were washed to remove the MS media, and the growth-promoting effects of bacterial VOCs were evaluated by considering shoot and root length, leaf number, ramification number, shoot fresh weight and root dry weight.

### 4.5. Biocontrol on Phytopathogenic Fungi under Salinity Conditions

*Bacterial suspension:* Cultures of *P. putida* strains were diluted in a sterile physiological solution to a final concentration of 10^9^ CFU/mL. Then, 100 μL of the resulting suspension was spread on Hoagland medium modified with 3% sucrose and 1.5% agar and allowed to grow for 24 h at 30 °C. The same procedure was carried out for control (with only100µL of sterile physiological solution, but without adding the strains). Fungal inoculum *Alternaria alternata* and *Sclerotium rolfsii* were used as fungal pathogens in the genus *Mentha*. These pathogens are known to cause economic losses by inflicting a heavy defoliation of the host, which affects both the yield and overall quality of mint oils [92,93]. *S. rolfsii* and *A. alternata* were grown on PDA medium (Britania^®^, Kolkata, India) in dishes for 5 and 7 days respectively. After this period, a disk of 5 mm diameter was obtained from the dishes and inoculated on sterile PDA dishes.

*Exposure to volatiles:* The bases of the Petri dishes, with 2 days of growth of bacteria and fungi, were inoculated and placed one against the other and sealed. By this method, *S. rolfsii* and *A. alternata* were exposed at 28 °C to bacterial VOCs without having any physical contact between them, and the diameter of the fungal colony was measured every day until days 4 and 6 of growth, respectively [94]. These experiments were repeated in triplicate, with five replicates being made for each treatment and control.

Hoagland medium (bacterial media) was supplemented with salt concentrations (a) 0 mM NaCl, (b) 75 mM NaCl, and (c) 100 mM NaCl and PDA media (fungal media), which in all cases were used as control conditions (0 mM NaCl). The fungal media were not supplemented with salt because fungi develop naturally on vegetal tissue.

### 4.6. GC–MS Profile of mVOCs

Bacteria were cultured in sealed 50 mL vials (sealed with metal crimp caps fitted with rubber septa to ensure that they were gastight) containing 10 mL of solid Hoagland medium with 0.7% agar and 3% sucrose, and subjected to different levels of stress (0, 75, 100 mM NaCl). These vials were inoculated with 500 μL suspension of the strains under study. All components were autoclaved individually prior to inoculation. The *E. coli* DH5α strain was included as a negative control. In addition, a control chromatographic profile was obtained from the culture medium, without bacterial growth, in order to identify the presence of volatile compounds in the selected system. Vials were placed in an oven at 28 °C for three days until carrying out the VOC analysis. Adsorption was programmed for 30 min at 40 °C, and SPME flex fibers (divinylbenzene/carboxen/Polydimethylsiloxane (DVB/CAR/PDMS) (Supelco) were desorbed at 210 °C for 1 min at the GC–MS injection port [65].

VOC analyses were performed on a Perkin Elmer Clarus 600 GC–MS using a DB5 column (60 m × 250 µm × 0.25 µm; J & W Scientific, Folsom, CA, USA) coupled to a mass analyzer with helium carrier gas at a constant flow rate of 1.0 mL/min. Volatiles absorbed by the SPME fiber were desorbed in the heated (250 °C) GC inlet for 60 s using splitless injection. The column temperature was programmed to start at 33 °C for 3 min, and heated at 10 °C/min to 180 °C and then at 40 °C/min from 180 to 220 °C (total run time = 18.7 min). Ions were generated using electron ionization (EI) (70 eV) and acquired at 4 scans/s over *m*/*z* 40–500. Volatile components were identified by comparison of retention times and recorded mass spectra against the NIST database, with an acceptance of similarity (SI) ≥ 800.

### 4.7. Statistical Analyses

Statistical analyses were performed using the Infostat software program version 2018. Normality and homoscedasticity of the data were first checked using the Shapiro–Wilk and Levene tests, respectively. Data were subjected to a two-way analysis of variance (ANOVA) (mVOcs × salt stress), with means being considered significant for *p* values < 0.05. This was followed by a comparison of multiple treatment levels with controls using Fisher’s post hoc LSD (least significant difference) test. The significance level for all calculations was set at *p* values < 0.05.

## 5. Conclusions

In conclusion, this study demonstrated a significant plant-growth-promoting effect of *P. putida* SJ46 and SJ04 VOCs on *M. piperita* under severe salt stress conditions. In addition, mVOCs emitted by both *P. putida* showed antagonism against the phytopathogen *A. alternata* under control and salt conditions, but only against *S. rolfsii* under control conditions. Through headspace sampling and GC–MS analyses, the mVOCs from both strains were found to be similar, but with the characteristic that the SJ04 strain emitted two compounds not found in the SJ46 blend. Salinity did not modify the compounds emitted by either strain. Moreover, our findings showed for the first time the differential effects of mVOCs emitted by rhizobacteria grown under different salt stress conditions, which may provide an effective approach for the development of microbial resources to help protect plants from salt stress conditions. However, further investigations on mVOC release by PGPR under salt stress conditions should now be carried out to try to elucidate the mode of action of specific compounds or mixtures that can regulate plant physiological processes and lead to salt stress tolerance as well as the control of *A. alternata* phytopathogens under these conditions. It is also necessary to consider performing future studies on technological improvements for bacterial VOC applications under greenhouse and open field conditions.

## Figures and Tables

**Table 1:** The effect on *M. piperita* of mVOCs emitted by native *P. putida* SJ04 and SJ46 plant-growth-promoting bacteria for different growth parameters under salt stress conditions (0 mM, 75 mM and 100 mM). Means followed by the same letter within a column are not significantly different according to Fisher’s LSD test (*p* < 0.05).

	Leaf nº	Node nº	Ramification nº	Shoot Length (cm)	Root Length (cm)
0 mM					
Non-exposed	25.36 ± 1.36 ^cde^	13.23 ± 0.60 ^cd^	1.86 ± 0.21 ^ab^	7.81 ± 0.85 ^d^	10.92 ± 0.51 ^b^
SJ46	27.33 ± 1.31 ^def^	14.00 ± 0.96 ^abc^	2.17 ± 0.27 ^a^	10.73 ± 0.92 ^e^	10.98 ± 1.11 ^b^
SJ04	29.70 ± 1.11 ^ef^	14.10 ± 0.35 ^cd^	2.00 ± 0.00 ^ab^	9.67 ± 0.86 ^e^	12.00 ± 1.64 ^b^
75 mM					
Non-exposed	18.00 ± 1.95 ^ab^	9.33 ± 0.96 ^ab^	1.78 ± 0.36 ^ab^	4.13 ± 0.45 ^bc^	5.07 ± 0.83 ^a^
SJ46	32.00 ± 2.74 ^f^	14.50 ± 2.37 ^d^	3.10 ± 0.50 ^cd^	3.60 ± 0.54 ^bc^	5.19 ± 1.36 ^a^
SJ04	20.00 ± 1.92 ^bc^	10.29 ± 1.08 ^d^	1.75 ± 0.25 ^ab^	4.89 ± 0.59 ^c^	4.90 ± 0.52 ^a^
100 mM					
Non-exposed	13.33 ± 2.01 ^a^	7.31 ± 1.15 ^a^	1.45 ± 0.21 ^a^	1.42 ± 0.16 ^a^	2.59 ± 0.56 ^a^
SJ46	22.83 ± 2.07 ^bcd^	13.40 ± 0.91 ^bcd^	2.40 ± 0.45 ^bcd^	3.56 ± 0.44 ^bc^	4.80 ± 0.49 ^a^
SJ04	23.60 ± 3.30 ^bcd^	12.50 ± 1.86 ^cd^	3.00 ± 0.33 ^d^	2.14 ± 0.27 ^ab^	4.65 ± 1.05 ^a^

**Table 2:** Identification of major chemical signals detected in mVOCs, based on GC–MS analysis and comparison with the NIST database, with acceptance of SI ≥ 800.

RT (min)	Compound	Non-Exposed			DH05			SJ04			SJ46		
		0 mM	75 mM	100 mM	0 mM	75 mM	100 mM	0 mM	75 mM	100 mM	0 mM	75 mM	100 mM
1.56	Nitrogen oxide	x	x	x	x	x	x	x	x	x	x	x	x
2.74	Trichloromethane	x	x	x	x	x	x	x	x	x	x	x	x
5.05	Propane, 2-chloro-2-nitro-	x	x	x	x	x	x	x	x	x	x	x	x
10.44	1-(N-phenylcarbamyl)-2 morpholino cyclohexene							x	x	x			
10.95	Tridecane							x	x	x			
11.49	n-Octanol							x	x	x	x	x	x
11.62	Decane							x	x	x	x	x	x
13.15	Butane, 2-methyl-				x	x	x	x	x	x	x	x	x
15.92	Tetradecane							x	x	x	x	x	x

## Data Availability

The data presented in this study are available on request from the corresponding author. The data are not publicly available due to privacy.

## Supplementary Materials

The following supporting information can be downloaded at: https://www.mdpi.com/article/10.3390/plants12071488/s1: Figure S1: Chromatograms of mVOCs produced by control media (not inoculated) under (a) 0, (b) 75 and, (c) 100 mM NaCl. Figure S2: Chromatograms of mVOCs produced by *E. coli* DH5α under (a) 0, (b) 75, and (c) 100 mM NaCl. Figure S3: Chromatograms of mVOCs produced by *P. putida* SJ46 under (a) 0, (b) 75, and (c) 100 mM NaCl. Figure S4: Chromatograms of mVOCs produced by *P. putida* SJ04 under (a) 0, (b) 75, and (c) 100 mM NaCl.
