# Supplementary figures and images for: Antifungal Activity and Alleviation of Salt Stress by Volatile Organic Compounds of Native Pseudomonas Obtained from Mentha piperita
Source: Plants (Basel). 2023 Mar 29;12(7):1488. doi: 10.3390/plants12071488 (PMC10097229; doi:10.3390/plants12071488)

29-Nov-2022 + 14:19:36  
Scan EI+  
TIC  
3.25e7

a)

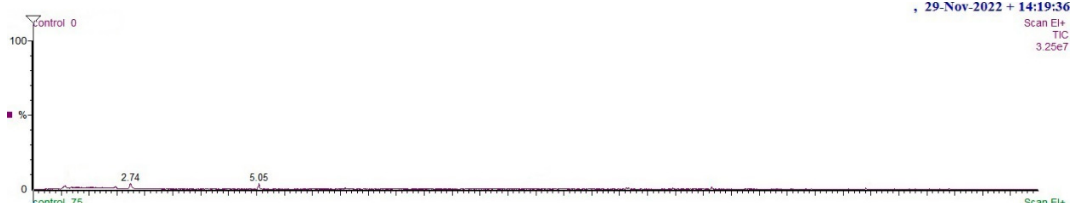

b)

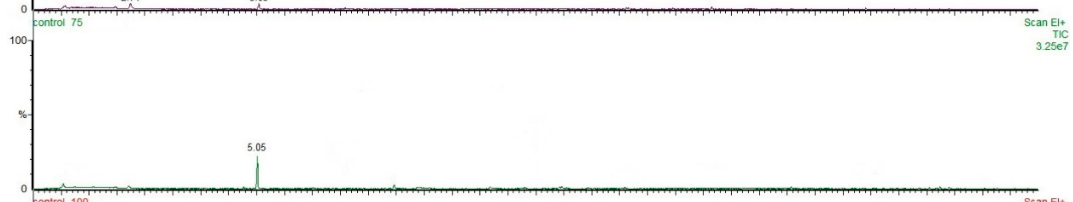

c)

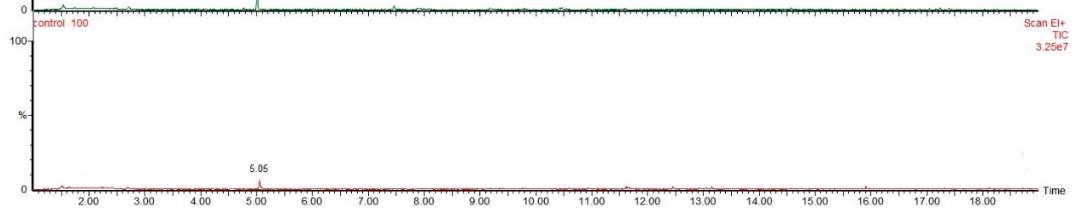

Supplement: Supplementary file 1 [file plants-12-01488-s001.zip › plants-2255163-SI/SF1.pdf]

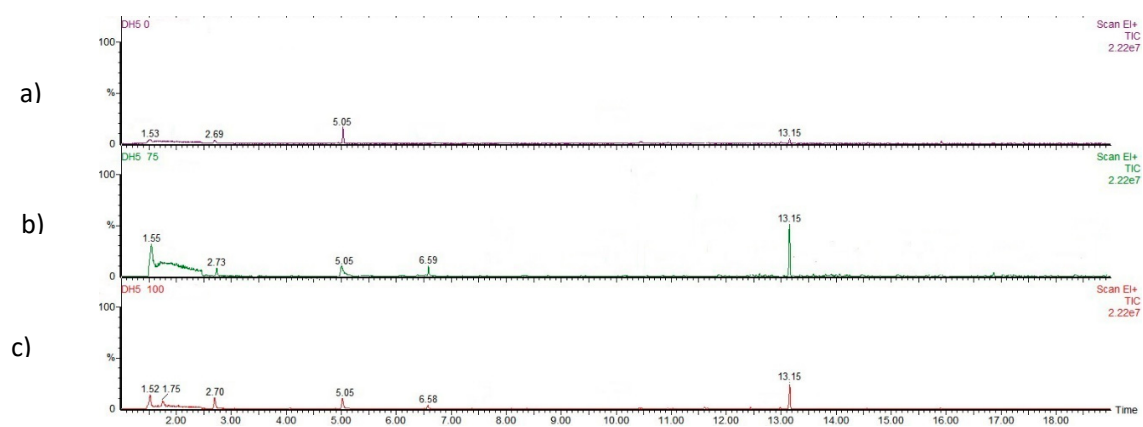

Supplement: Supplementary file 1 [file plants-12-01488-s001.zip › plants-2255163-SI/SF2.pdf]

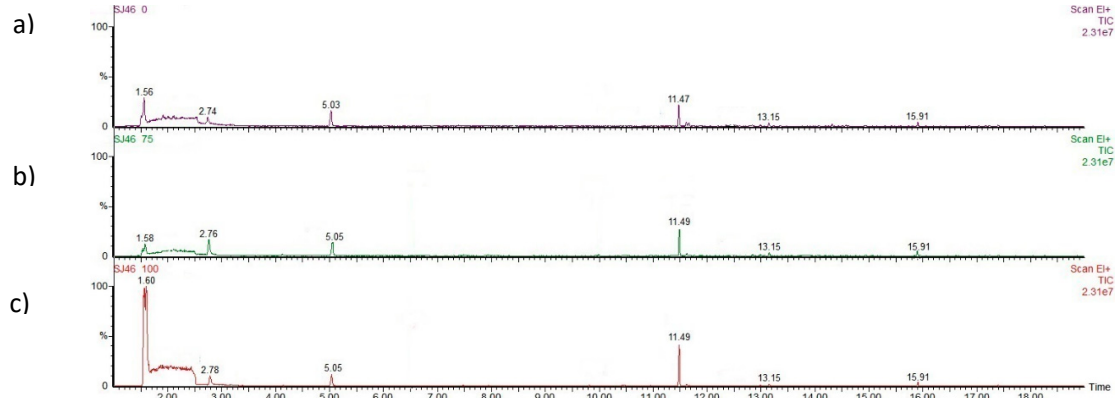

Supplement: Supplementary file 1 [file plants-12-01488-s001.zip › plants-2255163-SI/SF3.pdf]

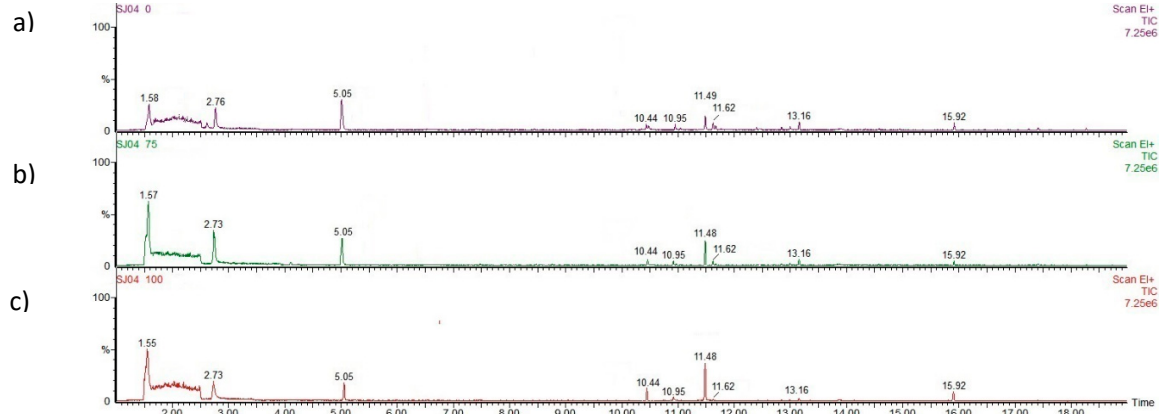

Supplement: Supplementary file 1 [file plants-12-01488-s001.zip › plants-2255163-SI/SF4.pdf]
